# Eating disorders in children: is avoidant-restrictive food intake disorder a feeding disorder or an eating disorder and what are the implications for treatment?

**DOI:** 10.12688/f1000research.13110.1

**Published:** 2018-01-18

**Authors:** Grace A. Kennedy, Madeline R. Wick, Pamela K. Keel

**Affiliations:** 1 Department of Psychology, Florida State University, 1107 West Call Street, Tallahassee, FL, 32304, USA

**Keywords:** eating disorders, Avoidant-Restrictive Food Intake Disorder, feeding disorders

## Abstract

Avoidant-restrictive food intake disorder (ARFID) is a current diagnosis in the “Feeding and Eating Disorders” section of the *Diagnostic and Statistical Manual of Mental Disorders* (fifth edition) and captures a heterogeneous presentation of eating disturbances. In recent years, ARFID has been studied primarily within the context of eating disorders despite having historical roots as a feeding disorder. The following review examines ARFID’s similarities with and differences from feeding disorders and eating disorders, focusing on research published within the last three years. Implications of this differentiation for treatment are discussed.

## Introduction and context

Avoidant-restrictive food intake disorder (ARFID) is a diagnosis in the “Feeding and Eating Disorders” section of the fifth edition of the *Diagnostic and Statistical Manual of Mental Disorders* (DSM-5) ^1^. ARFID is a reformulation of the DSM-IV diagnosis of feeding disorder of infancy or early childhood (FDIEC) ^2^ expanded to capture heterogeneous eating disturbances that lead to deficits in energy or nutritional intake. Reasons for insufficient intake include lack of appetite, the sensory characteristics of food, and fears related to the act of eating, such as fear of choking ^1^. These disturbances can confer harmful physiological or psychological consequences, including low weight or failure to grow, severe nutritional insufficiency, reliance on nutritional supplements, and psychosocial dysfunction ^1^.

Following ARFID’s introduction to the DSM-5 in 2013, an emerging line of research has involved descriptive studies comparing ARFID with eating disorders, such as anorexia nervosa (AN) and bulimia nervosa (BN) ^3– 5^. Furthermore, a plenary session on atypical eating disorders at the 2017 International Conference on Eating Disorders in Prague included two talks on ARFID ^6, 7^. This emerging body of work suggests that ARFID may be viewed as an eating disorder by the eating disorder research community. However, examination of ARFID’s history in the DSM along with the history of eating disorders in the DSM suggests that ARFID may represent a feeding disorder. Like children with ARFID, those with FDIEC displayed persistent failure to eat, leading to weight loss or failure to grow. The FDIEC diagnosis differed from the ARFID diagnosis in that FDIEC could be diagnosed only with an age of onset by six years old and did not account for other consequences of food restriction. Thus, the diagnosis of FDIEC was rarely used and research on its presentation was stagnant ^8^. Given the limited interest and utility of the diagnosis ^2, 8^, the DSM-5 Eating Disorders Work Group eliminated FDIEC and replaced it with ARFID, which includes all cases previously diagnosed with FDIEC along with cases that would not have met the age or weight consequences criteria ^2^.

Further supporting ARFID’s history as a feeding disorder, FDIEC fell under the umbrella of “Disorders Primarily Occurring in Infancy, Early Childhood, and Adolescence” in the DSM-IV, alongside pica and rumination disorder, in a section that was distinct from “Eating Disorders” ^9^. Interestingly, in the DSM-III-R, pica, rumination disorder, AN, and BN were all included under the umbrella of “Eating Disorders”, but it was noted that “Pica and Rumination Disorder of Infancy are […] probably unrelated to Anorexia Nervosa and Bulimia Nervosa” ( 10, page 65). This may have presaged the separation of AN and BN into their own separate section as “Eating Disorders” in the DSM-IV, suggesting that FDIEC, pica, and rumination disorder were all conceptualized as feeding disorders, even though only FDIEC was explicitly labeled as such. Moreover, the decision to unite ARFID, pica, and rumination disorder with AN, BN, and binge eating disorder in the DSM-5 in a section named “Feeding and Eating Disorders” suggests that some of these conditions are feeding disorders and some are eating disorders. Yet the DSM-5 provides no guidance on which diagnoses belong to which group.

According to the National Institute of Mental Health ^11^, “Eating disorders are [...] serious and often fatal illnesses that cause severe disturbances to a person’s eating behaviors. Obsessions with food, body weight, and shape may also signal an eating disorder”. No equivalent definition for “feeding disorder” is presented; however, Sharp *et al.* ^12^ defined feeding disorders as “severe disruptions in nutritional and caloric intake exceeding ordinary variations in hunger, food preference, and/or interest in eating” (page 116). Understanding ARFID’s place in the nomenclature is crucial for informing future research and treatment of the disorder. Thus, the following review examines the evidence for ARFID as a feeding disorder and as an eating disorder, focusing on research published within the last three years. We then discuss implications of this differentiation for treatment.

## Avoidant-restrictive food intake disorder as a feeding disorder

Within the past three years, ARFID has been studied largely within the context of eating disorders. Thus, ARFID’s similarity to posited feeding disorders, such as pica or rumination disorder, is discussed largely in terms of its dissimilarity to eating disorders. For instance, in a retrospective chart review of 7- to 17-year-olds admitted to a day program for eating disorders, Nicely *et al.* ^13^ found that compared to children with AN, BN, and other eating disorders, those with ARFID were more likely to have a comorbid diagnosis of autism spectrum disorder (ASD), learning disorder, or cognitive impairment ^13^. This is similar to findings primarily in pica in which common comorbidities are ASD or intellectual disability or both ^1^. Furthermore, individuals with ASD often demonstrate restrictive food selectivity due to sensory sensitivity to food texture and smell, which is consistent with ARFID ^14^. For some individuals with ASD, rigid food preferences may contribute to nutritional deficits and require clinical attention, warranting a separate diagnosis of ARFID. Indeed, Lucarelli *et al.* ^15^ presented a case study of a 4-year-old girl with ASD who presented to treatment for ARFID because of rigid food and meal rules ^15^. In this way, ARFID resembles other feeding disorders in which neurodevelopmental comorbidity may play a role in presentation. Yet this may not be unique to ARFID given growing research suggesting that individuals with AN are higher on autistic traits than healthy controls ^16, 17^. Thus, while ASD does have a high comorbidity with feeding problems related to sensory characteristics of food and rigid food preferences independent of weight concerns, the broader autistic phenotype may be over-represented in AN and ARFID, suggesting a potential link between ASD and restrictive eating and feeding disorders.

With regard to treatment, studies have repeatedly shown that children with ARFID enter care at a younger age and are less likely to be self-referred compared with children with eating disorders ^3, 4, 13, 18, 19^. In line with case reports, this suggests that ARFID onsets earlier and is more likely to be recognized as a problem by parents than are eating disorders ^15, 20, 21^. Indeed, numerous case reports and retrospective chart review studies have documented that individuals with ARFID enter care between the ages of 4 and 13 ^3, 4, 22^, suggesting similarity to the classic conceptualization of feeding disorders as a childhood problem ^9^.

In terms of clinical presentation, individuals with ARFID exhibit other notable differences from those with AN, BN, and their variants. By definition, ARFID may be diagnosed only in the absence of weight or shape concerns. Thus, as in pica and rumination disorder, the driving force behind feeding disturbances cannot be cognitive concerns related to the effects of food on appearance. In fact, previous research demonstrates that children presenting with ARFID often desire to gain weight rather than lose it ^19^. These findings may explain the multiple studies documenting a greater representation of males among those with ARFID than among those with AN or BN ^3, 4, 13^. It may also explain the greater use and acceptance of nasogastric feeding in ARFID compared with AN. Both Peebles *et al.* ^23^ and Ornstein *et al.* ^18^ have noted that children with ARFID require nasogastric feeding more often than those with AN but demonstrate less distress regarding this process. Patients with AN fear caloric content, which makes the route of caloric intake less relevant. In contrast, those with ARFID often fear choking during the process of eating but express notably less anxiety about taking in calories, making the route of intake highly relevant and the actual caloric content less relevant ^18, 23^.

Taken together, these data suggest that ARFID is distinct from eating disorders on patterns of comorbidity, age of presentation, referral source, gender distribution, and response to intervention modalities in ways that make it more similar to pica and rumination disorder. This conclusion should be interpreted with some caution, however, given the paucity of research on pica or rumination disorder or research comparing these conditions with ARFID.

## Avoidant-restrictive food intake disorder as an eating disorder

With regard to similarities to eating disorders, characteristics shared with AN have been identified. Like AN, ARFID often presents with low weight and malnutrition, thus requiring a multidisciplinary team, including family members, physicians, mental health professionals, and dietitians, to restore weight. Of note, a multidisciplinary team of providers and family members has been presented as the best practice for all feeding disorders ^12^, highlighting a broader overlap between feeding and eating disorders when they occur in children. For instance, Peebles *et al.* ^23^ described an inpatient refeeding protocol that could be employed for children and adolescents with either AN or ARFID ^23^. This protocol used a meal plan with increasingly higher caloric content and heavy involvement of parents in the refeeding process both during inpatient hospitalization and in outpatient aftercare. Furthermore, Forman *et al.* ^4^ found that treatment of ARFID did not differ from that of AN or atypical AN in a large retrospective chart review of 700 adolescents attending eating disorder programs across 14 different sites ^4^. Importantly, however, treatments differed considerably by site, such that site, rather than diagnosis, may have been the main determinant of what treatments were offered. Furthermore, at one-year follow-up, AN and ARFID did not differ significantly in likelihood of median body mass index being at least 90% expected; however, this may have reflected limited statistical power, as this outcome was twice as likely for AN patients compared with ARFID patients. Nonetheless, treatments of ARFID and AN in children share components because of shared medical concerns and utility of involving parents in the care of their child’s illness.

Much like children and adolescents with AN, those with ARFID present with strict food rules about what they can and cannot eat ^15, 21^. Food exposure to reduce avoidance of feared foods is used in the treatment of both AN and ARFID ^23^. Furthermore, both retrospective chart reviews and case reports illustrate that some individuals with ARFID transition to a diagnosis of AN once weight is partially restored ^22, 24^. In a chart review of 205 patients presenting to care for an eating disorder, Norris *et al.* ^24^ found that 12% of patients with ARFID transitioned to a diagnosis of AN during treatment, suggesting that a diagnosis of ARFID may serve as a risk factor for the development of AN. Case reports presented by Maertens *et al.* ^22^ suggest that weight concerns may emerge after refeeding in some individuals with comorbid ARFID and obsessive-compulsive disorder, resulting in the later diagnosis of AN that was not evident when patients were very underweight.

Another consideration is the overlap between ARFID and non-fat phobic AN (NFP-AN). Individuals with NFP-AN restrict energy intake, resulting in low weight; however, they deny fear of fat or weight gain. In a review on NFP-AN, Becker *et al.* ^25^ described cases whereby restriction of food intake was due to religiosity and gastrointestinal discomfort as well as a lack of appetite. Given that DSM-5 AN can be diagnosed in an individual who engages in behaviors to prevent weight gain and does not recognize the seriousness of low weight but reports no other body image disturbance, some cases of ARFID bear a strong resemblance to NFP-AN. Unlike those with ARFID, however, those with NFP-AN are averse to weight gain, which is an important differentiation between the two.

In summary, despite ARFID’s differentiations from eating disorders, it demonstrates key similarities to AN in regard to presenting symptoms of underweight and rigid food rules, potential to transition to endorsing concerns about shape and weight as motivating food restriction, and treatment. These similarities may explain why ARFID has been compared with eating disorders in several recent studies and why it was included in talks focusing on atypical eating disorders.

## Conclusions and future directions

Research on ARFID represents an emerging field of study. ARFID resembles a feeding disorder in demographic features, comorbidity, source of presentation, and greater acceptance of invasive treatments. Conversely, ARFID resembles AN in children in terms of management and treatment of the illness and shares similar presentations with NFP-AN in some cases. Moreover, ARFID appears to precede AN in a substantial minority (12%) of patients with ARFID. Thus, rather than revealing whether ARFID is a feeding disorder or an eating disorder, this review suggests ways in which ARFID may be conceptualized as *both* a feeding and an eating disorder and proposes that the similarity and emphasis depend on clinical presentation. Indeed, those with ARFID who present with sensory sensitivities (for example, rejecting food on the basis of color or texture) may have more in common with pica or rumination disorder, as sensory stimulation may contribute to aberrant eating behaviors in all three disorders ^26, 27^. It is possible that underlying deficits in sensation processes are at play in pica and rumination disorder and, in a subset of cases, ARFID. In contrast, ARFID associated with intense fear of vomiting and gastrointestinal distress may have greater similarity to the conceptualization of NFP-AN. Migration from ARFID to AN underscores the fluidity of the distinction between ARFID and AN, which may reflect developmental shifts as well as changes in weight over the course of development and treatment.

Given ARFID’s history as a feeding disorder, it is surprising that the focus of recent research has been primarily within the field of eating disorders; however, there are exceptions to this. Sharp *et al.* ^12^ suggested that ARFID is the psychiatric disorder that best captures feeding disorder presentations and offered a review of studies on techniques for treating ARFID-like feeding disturbances in children. Furthermore, in addition to being referred to eating disorder programs, patients with ARFID are referred to feeding disorder programs and gastroenterology clinics ^28, 29^. Thus, greater communication between those who approach ARFID from a feeding disorder perspective and those who approach ARFID from a eating disorder perspective may be beneficial in both the treatment and the understanding of ARFID. Indeed, to this end, Thomas *et al.* ^30^ have drawn on the knowledge base of feeding and eating disorders to put forth a conceptual neurobiological model of ARFID and a potential avenue for treatment enhancement.

In summary, ARFID possesses characteristics relevant to both feeding and eating disorders and may represent the missing link between these differing types of disorders. As such, research on ARFID may yield insights into underlying mechanisms contributing to disturbances in eating across the range of these disorders. More information on ARFID from both feeding and eating disorder perspectives could improve greater understanding of not only ARFID but also additional feeding or eating disorders to which ARFID has similarities.
